# Immune mechanisms of cardiac aging

**DOI:** 10.20517/jca.2023.02

**Published:** 2023-03-09

**Authors:** Daniel R. Goldstein, Ahmed Abdel-Latif

**Affiliations:** 1 Department of Internal Medicine, University of Michigan, Ann Arbor, MI 48109, USA.; 2 Department of Microbiology and Immunology, University of Michigan, Ann Arbor, MI 48109, USA.; 3 Division of Cardiovascular Medicine, Department of Internal Medicine CVC, University of Michigan, Ann Arbor, MI 48109, USA.; 4 Ann Arbor VA Healthcare System, Ann Arbor, MI 48109, USA.

**Keywords:** Aging, cardiac fibrosis, heart failure with preserved ejection fraction

## Abstract

Advances in healthcare and improvements in living conditions have led to rising life expectancy worldwide. Aging is associated with excessive oxidative stress, a chronic inflammatory state, and limited tissue healing, all of which result in an increased risk of heart failure. In fact, the prevalence of heart failure approaches 40% in the ninth decade of life, with the majority of these cases suffering from heart failure with preserved ejection fraction (HFpEF). In cardiomyocytes (CMs), age-related mitochondrial dysfunction results in disrupted calcium signaling and covalent protein-linked aggregates, which cause cardiomyocyte functional disturbances, resulting in increased stiffness and diastolic dysfunction. Importantly, aging is also associated with chronic low-grade, sterile inflammation, which alters the function of interstitial cardiac cells and leads to cardiac fibrosis. Taken together, cardiac aging is associated with cellular, structural, and functional changes in the heart that contribute to the rising prevalence of heart failure in older people.

## Graphical Abstract

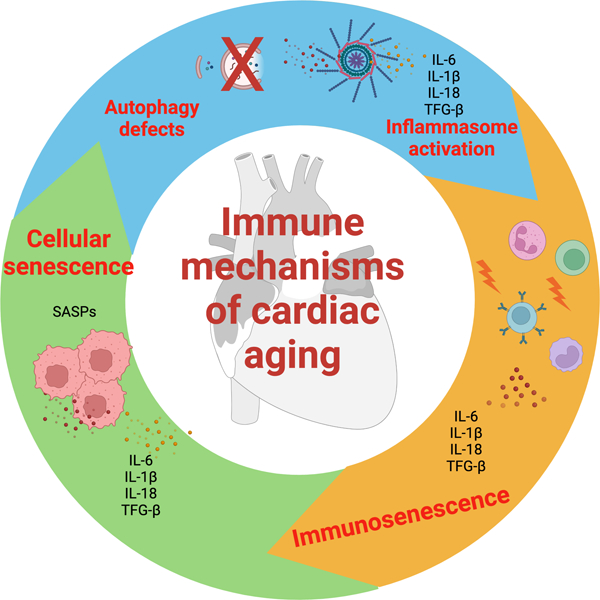

## INTRODUCTION

The percentage of people above the age of 65 increased to 16.9% in 2020, and this figure is expected to reach 22% by 2050. As the population ages, there is an increasing focus on understanding cardiac pathophysiological changes associated with aging. These changes include an increased risk of cardiovascular disease, heart failure, and changes in the structure and function of the heart. Additionally, the aging process can lead to changes in the heart’s electrical conduction system, leading to arrhythmias. As such, there is a need for further research into the mechanisms underlying these changes and potential interventions to reduce the risk of cardiovascular disease in older people. Aging is associated with a chronic low-grade inflammation, termed inflammaging, which describes the normal aging process and the associated increased risk of age-related diseases. It is characterized by a persistent low-grade inflammatory response that can be caused by a variety of factors, including cellular senescence, immune system dysfunction, and increased organ stress. The effects of inflammaging on the heart are far-reaching and may include an increased risk of cardiovascular diseases, including coronary artery disease, stroke, and heart failure. This review discusses major pathophysiological pathways that lead to cardiac aging, focusing on the role of inflammaging.

## CARDIAC FUNCTIONAL AND STRUCTURAL CHANGES DURING AGING

Aging is associated with a constellation of left ventricular (LV) hypertrophy, left atrial enlargement, and interstitial fibrosis with diastolic dysfunction, with no changes in systolic function. These structural changes are characteristic of HFpEF and are typically associated with a significant reduction in exercise capacity. Age-specific cardiac imaging studies documented an increased prevalence of diastolic dysfunction with aging. Cardiac magnetic resonance (CMR) studies documented a significant 5 mg/mL/year increase in LV mass/volume ratio with a corresponding 0.4 mL/year reduction in stroke volume owing to the thickened cardiac muscle dysregulation^[1]^. Echocardiographic studies reached similar conclusions and demonstrated that the prevalence of diastolic dysfunction increased exponentially with age^[1]^. While echocardiographic parameters of diastolic dysfunction were rarely observed in individuals younger than 50, over half of the individuals between 70 and 80 years of age had echocardiographic evidence of diastolic dysfunction. This prevalence rises to two-thirds of study participants older than 80 years of age^[2]^.

Cardiac aging is also associated with electrophysiological changes that result in decreased intrinsic heart rate, response to adrenergic signaling, and increased arrhythmogenic effects^[3]^. Combined with diastolic dysfunction seen in aging, which reduces ventricular compliance and stroke volume, reduced intrinsic heart rate with aging results in diminished cardiac output. Furthermore, alterations to adrenergic and cholinergic signaling within CMs result in their hypertrophy as well as cardiac fibrosis, contributing to age-related structural and functional changes^[4,5]^. Combined with the limited ability of the heart muscle to regenerate, excessive cellular loss contributes to cardiac dysfunction in aging. Therefore, structural changes in the heart with aging have been linked to the development of heart failure, particularly with preserved ejection fraction.

In addition to changes in the ventricles, aging is associated with important structural and functional changes affecting the atria. Aging-associated oxidative stress, inflammation, cellular senescence, and extracellular matrix remodeling increase atrial fibrosis resulting in a decrease in contractility and conduction velocity^[6,7]^. Atrial fibrosis can then impair atrial electrical conduction and mechanical function, creating a substrate for atrial fibrillation (AF) initiation and maintenance and the associated risk of embolic stroke.

## CELLULAR PATHWAYS ASSOCIATED WITH INFLAMMAGING

Aging is associated with a chronic low-grade sterile inflammatory state, termed “inflammaging”, which is one of the hallmark features of aging and can be linked to increased morbidity and mortality in older individuals^[8]^. Sterile inflammation is a type of inflammation that is triggered by non-infectious stimuli, such as increased cellular senescence, immunosenescence, dysregulated inflammasome activation, mitochondrial dysfunction, age-related autophagy and mitophagy defects^[9]^, and systemic predisposing factors such as changes in the number and composition of intestinal microbiota^[10,11]^ and clonal hematopoiesis of indeterminate potential (CHIP)^[12]^. Sterile inflammation involves the activation of innate immune cells, such as macrophages and neutrophils, by damage-associated molecular patterns (DAMPs) that bind to pattern recognition receptors (PRRs) on immune cells. Sterile inflammation can have beneficial effects, such as promoting wound healing and tissue repair, but it can also have harmful effects, such as causing chronic diseases, organ damage, or autoimmunity. Consequently, multiple studies have documented increased levels of inflammatory cytokines in the peripheral blood of healthy older individuals, even in the absence of underlying stress or infection. The risk and consequences of this inflammatory response affect the heart. There is evidence of increased activation of inflammatory pathways, e.g., increased levels of nuclear factor kappa-light-chain-enhancer of activated B cells (NF-κB), which could lead to the production of pro-inflammatory cytokines, and pro-inflammatory macrophages in older hearts, particularly in females^[13–15]^.

Mechanistically, mitochondrial dysfunction plays a central role in the aging process and is at the crossroads of central pathways involved in inflammaging^[16–18]^. Aging is associated with mitochondrial structural abnormalities resulting in functional deterioration and increased reactive oxygen species (ROS) generation. In addition, aging is associated with reduced activation of 5’ AMP-activated protein kinase (AMPK) and sirtuins (SIRT), among other key regulators of mitochondrial function, cellular energy balance, and homeostasis^[19]^. Indeed, animal models of cardiac hypertrophy documented the link between a reduction in SIRT6 expression and the development of hypertrophy and diastolic dysfunction^[20]^. Additionally, the expression of SIRT3, an essential regulator of mitochondrial function, is reduced with aging, resulting in endothelial cell dysfunction and diastolic dysfunction^[21]^. Furthermore, the downregulation of AMPK and sirtuins results in a shift of mitochondrial oxidation favoring glycolytic pathways, which promotes inflammation in multiple cell lines, including coronary endothelial cells^[22,23]^. In addition to the effects of AMPK and sirtuins on the mitochondria and energy homeostasis, these pathways play a critical role in modulating NF-κB activation, and the downregulation of both these pathways results in uninhibited NF-κB activity and exacerbates inflammatory responses^[15,24]^. Mitochondrial dysfunction and disturbances in cellular homeostasis result in the release of mitochondrial DNA (mtDNA), eventually leading to increased inflammation through the activation of toll-like receptors (TLRs) and the nucleotide-binding oligomerization domain-3 (NLRP3) inflammasome. Beyond the role of mitochondrial dysfunction in inflammation, mitochondrial dysfunction leads to disturbed calcium signaling due to changes in the type 2 ryanodine receptor (RyR2) and the sarcoplasmic reticulum Ca^2+^ ATPase pump (SERCA), thus contributing to cardiomyocyte dysfunction and diastolic heart failure.

**Figure 1.**
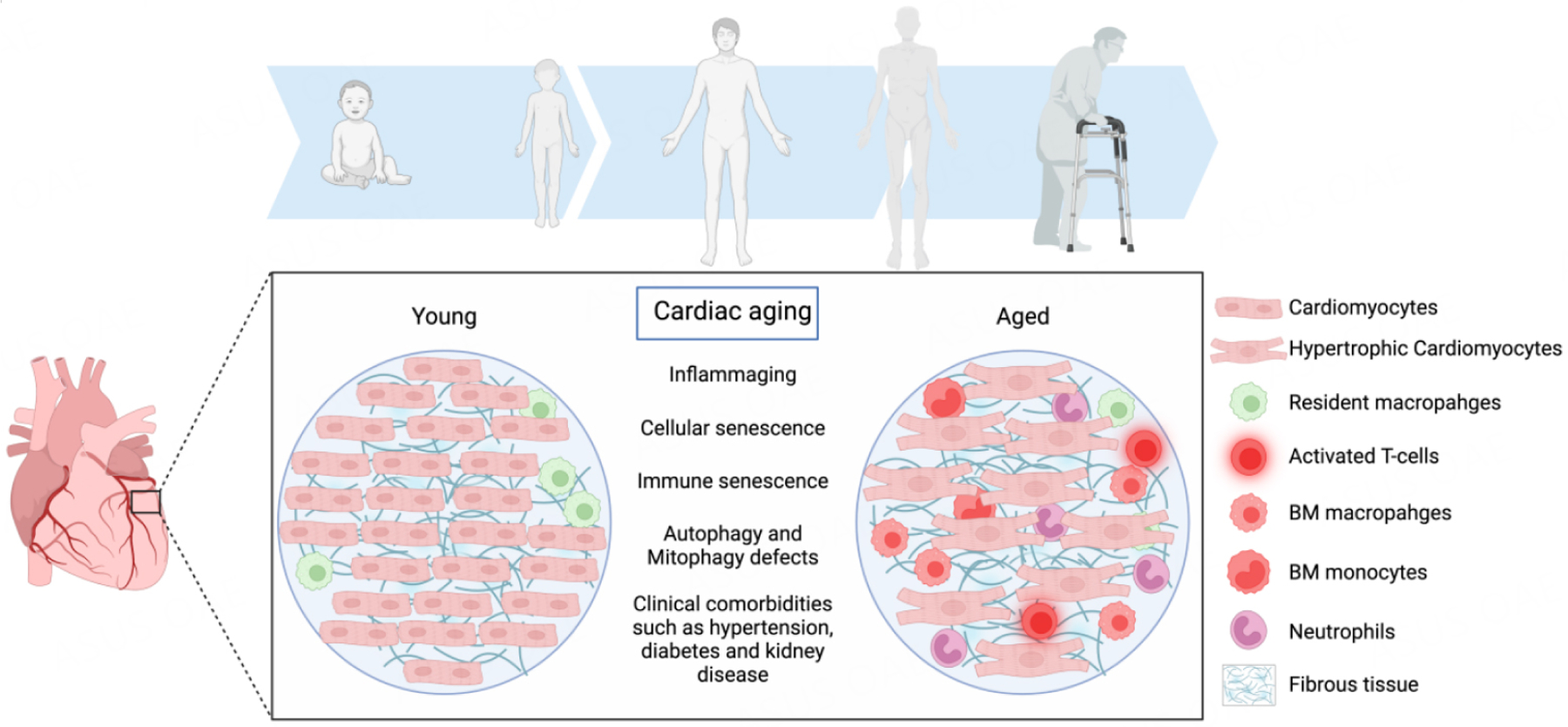
Cardiac aging is associated with a myriad of cellular and structural changes that result in heightened systemic and cardiac inflammation. These changes cause a shift in cardiac macrophage populations with an abundance of bone marrow-derived, pro-inflammatory macrophages and a reduction in the percentage of resident anti-inflammatory macrophages. The heart also becomes populated with other immune cells that promote tissue inflammation, such as monocytes, neutrophils, and T cells. This phenomenon is associated with cardiac fibroblast activation and fibrosis. Additionally, aging is associated with cardiomyocyte hypertrophy and increased stiffness. Taken together, aging-related changes in the heart result in diastolic dysfunction and the development of heart failure with preserved ejection fraction (the figure was prepared using Biorender, https://biorender.com/).

Aging is associated with a plethora of pathophysiological changes with a resulting chronic low-grade inflammatory state [Figure 1]. Cellular senescence, a phenomenon exacerbated by aging, is caused by cellular stresses such as telomere shortening and leads to the release of pro-inflammatory cytokines such as interleukin (IL)-6, IL-1β, and IL-1α^[9]^. The senescence-associated secretory phenotype (SASP) is known to be important for the clearance of senescent cells. However, aging of the immune system can impair its efficiency in clearing senescent cells, exacerbating the pro-inflammatory state^[8]^. Age-related changes in the innate immune system can reduce phagocyte capability, respiratory burst, and function of natural killer (NK) and dendritic cells, thus impairing immune cell functions and their interactions^[8]^. Increased production of ROS, generated by the aging process, overwhelms the scavenger systems and leads to the production of damage-associated molecular patterns (DAMPs), activation of the cyclic GMP-AMP synthase (cGAS)-stimulator of interferon genes (STING) (cGAS-STING) pathway, and stimulation of the NLRP3 inflammasome. This results in an extensive inflammatory response. Moreover, telomere shortening is associated with mitochondrial dysfunction due to decreased expression of peroxisome proliferator-activated receptor coactivator-1α and −1β. All of these age-related pathophysiological changes collectively lead to a chronic low-inflammatory state that affects both the innate and adaptive immune systems^[25]^.

## AGE-ASSOCIATED IMMUNE CELL CHANGES AND THEIR EFFECTS ON THE HEART

Aging is associated with a dysregulated innate immunity in both animals and humans. The hallmarks of these phenotypic changes are a heightened baseline inflammatory state and an impaired immune response to external pathogens and stimuli. Mechanistically, this phenomenon is linked to altered myeloid cell production and activation, impaired expression, and activation of PRRs. Collectively, these factors result in a dysregulated immune system, increased cytokine production, and an increased inflammatory state with aging^[26]^.

Significant reduction in the proliferative capacity of hematopoietic progenitor cells in the bone marrow results in the skewed composition of the myeloid cell compartment^[27]^. In addition to changes in the number of circulating and tissue-bound myeloid cells, studies have demonstrated functional impairment with aging, including reduced chemotaxis, migration, and phagocytosis, as seen among aging neutrophils^[28]^. Furthermore, a dysregulated neutrophil response to stimuli and reduction in the production of anti-inflammatory and anti-apoptotic cytokines exacerbate the baseline inflammatory state^[29]^. These changes also extend to natural killer (NK) cells, monocytes, and macrophages during aging. NK cells show a reduction in both cytotoxic and secretory functions with aging, resulting in impaired response to pathogens and dysregulation of immune signaling with other immune cells^[30]^. Similarly, monocytes and macrophages demonstrate a reduced response to TLRs and cytokine signaling with aging concomitant with inappropriately increased cytokine production^[31,32]^. Macrophages play a significant role in the development of HFpEF, as they can produce and secrete various pro-inflammatory and pro-fibrotic cytokines, which can lead to the remodeling of the left ventricle^[33]^. This remodeling can lead to adverse changes in the heart’s contractile and diastolic functions, which can ultimately lead to various forms of heart failure. Additionally, dysregulated macrophages can contribute to other processes that are known to contribute to HFpEF, such as oxidative stress, fibrosis, and vascular stiffening^[34]^. Therefore, pro-inflammatory macrophages may be an important target for therapeutic interventions in HFpEF. Taken together, aging is associated with dysregulated innate immune system, impaired response to external stimuli, and exacerbated cytokine production resulting in heightened baseline inflammation.

While macrophages are believed to be the predominant immune cell population in the heart during physiological conditions, recent studies demonstrate that additional leucocyte populations populate the heart in the absence of injury. Among these populations, T cells demonstrate significant dynamic changes with aging^[35]^. Structural changes seen with aging have been correlated with the accumulation of activated CD4^+^/Foxp3^−^/IFNγ^+^ T cells in the mediastinal lymph nodes draining the heart. The shift in T cell populations contributes to age-related cardiac inflammation and dysfunction. Indeed, adoptive transplantation studies of mediastinal lymph node T cells from aged, but not young, mice demonstrated primed activation and cardiotropism, resulting in a mild form of cardiac dysfunction^[35]^. This study suggests that the contribution of T cells to aging-related cardiac dysfunction is a result of direct effects as well as indirect effects through the regulation of the immune system. The mechanisms behind priming T cells during the cardiac aging process are not fully understood but could be related to exposure to cardiomyocyte components that serve as auto-antigens, which results in an autoreactive immune response^[35,36]^. In fact, studies have confirmed the role of MHCII^+^ resident macrophages in presenting CM components to T cells resulting in priming them and a consistent population of T- and B-cells in the heart in the absence of injury^[37]^. Aging also affects the adaptive immune system through reduced production of naïve T cells and Treg cells, resulting in an imbalance towards a higher percentage of self-reacting T cells with an increased incidence of auto-immune diseases^[36]^. Similarly, reduction in B-cells, especially those expressing CD28, results in an imbalance between regulatory and pro-inflammatory T cells^[38,39]^. Collectively, changes in the innate and adaptive immune systems potentiate chronic inflammation in the heart with aging and subsequently contribute to functional deterioration.

## HEART FAILURE WITH PRESERVED EJECTION (HFPEF) IN THE AGING POPULATION

**Figure 2.**
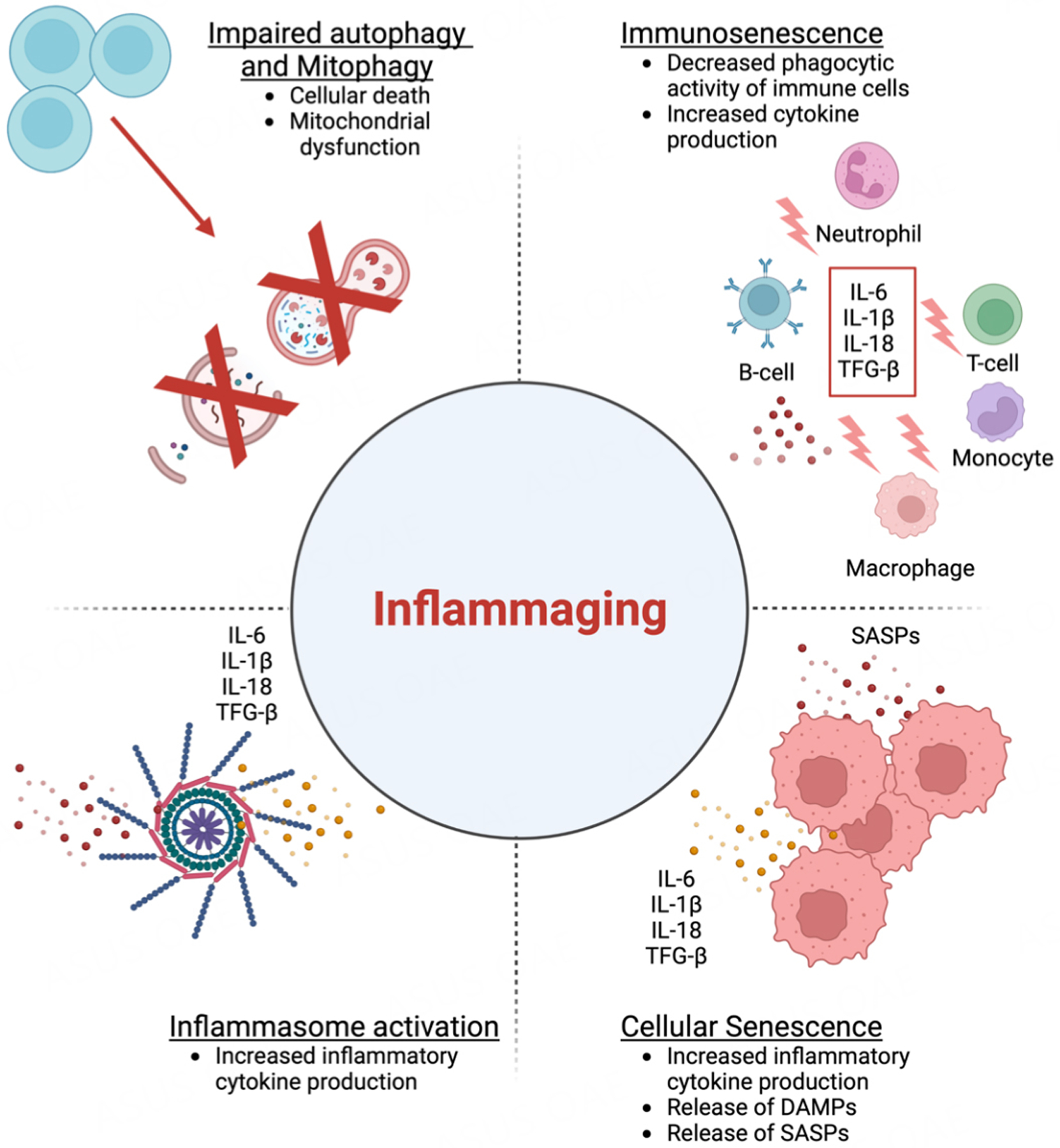
The aging process has a complex impact on the immune system, leading to sustained low-grade immune activation, reduced sensitivity to immunogenic stimuli, and accumulation of senescent cells. These changes result in a chronic inflammatory process known as SASP (senescence-associated secretory phenotype). In addition, increased inflammation with aging may be attributed to inflammasome activation, due to defects in autophagy and mitophagy. This pro-inflammatory milieu perpetuates the chronic low-grade inflammation commonly seen in aging. This figure was modified from Puspitasari *et al.*^[69]^. Additionally, the figure was prepared using Biorender, https://biorender.com/).

The prevalence of HFpEF has risen in the population over the last two decades. This could be attributed to the increasing prevalence of its risk factors, such as aging, obesity, hypertension, and type 2 diabetes mellitus (T2D). In particular, aging is associated with tissue fibrosis, a process that is linked to activated cardiac fibroblasts (CFs), neurohormonal activation, and heightened inflammation [Figure 2]. Multiple age-related cellular pathways that result in positive oxidative stress led to increased ECM production and significant impairment of its clearance, thus tipping the proteolytic balance towards net ECM accumulation, cross-linking, and increased myocardial stiffness. The Angiotensin II (Ang II) system is a key neurohormonal pathway activated in aging that has a significant role in cardiac inflammation through direct and indirect pathways. Ang II binds to angiotensin 1 receptors (AT1) and directly activates cardiac fibroblasts resulting in increased production of extracellular matrix components, collagen deposition in the extracellular space, and cardiac fibrosis. Beyond its direct effects on CFs, Ang II-AT1 signaling promotes inflammation through increased intracellular ROS, mitochondrial dysfunction, and the release of mtDNA. The combination of the direct and indirect effects of AngII-AT1 signaling in aging results in diastolic dysfunction and a higher prevalence of HFpEF^[40]^.

In addition to HFpEF due to increased cardiac fibrosis, structural cardiac changes can be caused by infiltrating cardiac disease. Autopsy studies have demonstrated a high prevalence of amyloidosis in older people, with the acquired wild-type variant (ATTRwt) being the most common aging-related variant^[41]^. Mechanistically, aging could destabilize transthyretin through alterations in post-transcriptional changes in the protein or its chaperones^[42,43]^. Amyloidosis is caused by the deposition of amyloid fibrils in the ECM, resulting in increased wall thickness, increased stiffness, and reduction of contractile function in the long term.

The effects of structural cardiac changes leading to diastolic and systolic dysfunction are exacerbated by the high prevalence of comorbidities in the aging population. These comorbidities profoundly affect cardiac function and structure and include a sedentary lifestyle, hypertension, diabetes, and atrial fibrillation. Among the common comorbidities seen in aging, ischemic heart disease represents a major cause of structural heart disease in older people. Studies have documented a prevalence of obstructive heart disease as high as 60% in individuals over the age of 60^[44]^. Furthermore, studies have documented poor healing response after myocardial infarction in older people with worse adverse cardiac remodeling and rapid progression to systolic heart failure^[45]^. In addition, obesity is also prevalent in older people in the US and contributes to the heightened inflammatory state, renin-angiotensin system activation, increased Ang II production, and the development of diastolic heart failure^[34,46]^.

## THERAPEUTIC AND TRANSLATIONAL PERSPECTIVES IN CARDIAC INFLAMMAGING

Mitochondrial dysfunction and reduction in AMPK signaling play a critical role in inflammaging through the release of ROS and mtDNA, which activates the innate immune system and contributes to the chronic inflammatory response. Therefore, multiple studies have explored the therapeutic potential of drugs that maintain mitochondrial function during aging. Metformin reduces oxidative stress through the inhibition of mitochondrial complex I, resulting in the activation of AMPK and improved mitochondrial function^[47]^. Nutraceuticals, such as resveratrol, attenuate mitochondrial-induced inflammaging and endothelial cell dysfunction by activating cAMP-AMPK^[48]^. Given the detrimental impact of oxidative stress on mitochondrial function, antioxidants such as Coenzyme Q have been proposed as therapeutic alternatives. Clinical trials have shown modest benefits for such approaches in chronic heart failure patients, which could be explained, at least in part, by the lack of specificity to mitochondria^[49]^. Recent approaches that utilize triphenylphosphonium as a mitochondria-targeted vehicle are being tested in clinical trials^[50]^. Given the role of senescence in the age-related inflammatory response, senolytic therapies have been proposed as a therapeutic target. Dasatinib, a tyrosine kinase inhibitor, combined with Quercetin, a natural flavonoid with senolytic activity, reduces the effect of aging on vascular function and specifically reduces vascular calcification^[51]^. Similarly, Navitoclax, which inhibits the apoptosis regulator protein B-cell lymphoma 2 (Bcl-2), improves cardiac function and survival in aged mice^[52]^. Many of these approaches await safety and large efficacy clinical studies.

Inflammation plays a critical role in the pathophysiology of cardiac dysfunction with aging. Accordingly, targeting inflammation represents a promising new strategy for the management of age-related cardiovascular diseases. Among the various pathways targeted in clinical studies, the inhibition of NLRP3 inflammasome and the downstream IL-1β signaling appear to be the most effective targets. Colchicine, which inhibits tubulin polymerization and the assembly of the multimeric NLRP3 inflammasome, reduces major cardiovascular events (MACE) in patients with chronic stable coronary artery disease^[53,54]^ as well as those with recent myocardial infarction^[55]^. However, colchicine did not improve clinical outcomes when administered immediately after myocardial infarction suggesting patient- and clinical-scenario-specific benefits. NLRP3 activation results in the release and activation of IL-1β and IL-6, major pro-inflammatory cytokines.

The CANTOS trial, a landmark randomized controlled clinical trial, found that canakinumab, a monoclonal antibody directed against interleukin-1β, significantly reduced the risk of MACE in patients with a history of myocardial infarction and elevated levels of high-sensitivity C-reactive protein (hs-CRP). The trial included 10,061 participants from 33 countries with a median follow-up of 3.7 years. It demonstrated a 15% reduction in all-cause mortality, a 16% reduction in MACE, and a 25% reduction in inflammatory markers. Additionally, canakinumab reduced hs-CRP levels and the risk of all-cause mortality among patients with elevated CRP^[56]^. Similar findings were observed among patients with recent myocardial infarction^[57,58]^. In the VCU-ART pilot studies, forty patients with STEMI who underwent fourteen days of anakinra treatment showed a reduction in the area under the curve for hs-CRP, and a signal for reduced progression to HF when compared to placebo^[59–61]^. The MRC-ILA-Heart study of 182 patients with non-ST-segment elevation myocardial infarction (NSTEMI) revealed that IL-1 blockade with anakinra successfully reduced CRP levels at 7 days post-NSTEMI yet failed to yield any improvement in clinical outcomes^[62]^. Given that the majority of benefits from Canakinumab is in patients with documented inflammation and high risk of cardiovascular events, some experts suggest that canakinumab may be considered for patients with prior myocardial infarction, high-sensitivity C-reactive protein levels of 2 mg/L or greater, and low-density lipoprotein cholesterol levels below 70 mg/dL, who are at high risk of recurrent events despite optimal medical therapy. However, this recommendation is not widely accepted, and more evidence of cost-effectiveness is needed to support it due to the high cost of Canakinumab.

Other targetable cytokines in the systemic inflammatory response include IL-6. The RESCUE trial was a phase 2 trial that enrolled 264 patients with moderate to severe chronic kidney disease and high-sensitivity CRP of at least 2 mg/L^[2]^. Patients were randomly assigned to receive a placebo or ziltivekimab, an IL-6 ligand monoclonal antibody, at 7.5 mg, 15 mg, or 30 mg every 4 weeks up to 24 weeks^[63]^. The primary endpoint was a change in high-sensitivity CRP from baseline to week 12. The secondary endpoints included changes in other inflammatory and thrombotic biomarkers, such as fibrinogen, D-dimer, IL-6, and sgp130. In this study, ziltivekimab significantly reduced multiple biomarkers of systemic inflammation and the magnitude of change in high-sensitivity CRP with ziltivekimab was nearly twice as large in RESCUE as in the recent CANTOS trial of canakinumab. The ZEUS study is a phase 3 trial that is currently enrolling patients with chronic kidney disease and elevated high-sensitivity CRP to test whether ziltivekimab can reduce cardiovascular events. The primary endpoint is a composite of cardiovascular death, nonfatal myocardial infarction, or nonfatal stroke and the results are expected in 2024 (https://clinicaltrials.gov/).

A word of caution is necessary when targeting inflammation, particularly in older people with a dysregulated immune system. Many of these therapies result in an increased risk of infections and, in the case of colchicine, increased non-cardiac death. This highlights the need for more targeted immunomodulatory therapies with fewer immunosuppressive effects.

Multiple nonpharmacological approaches have been adopted to mitigate the effects of aging on the cardiovascular system. Nonpharmacological approaches using CAR T cells that target senescent cells are demonstrating promise in pre-clinical and early clinical studies^[64]^. Recent studies have demonstrated that regular exercise can help reduce inflammation associated with aging^[65]^. Physical activity was associated with lower circulating markers of inflammation such as pro-inflammatory cytokines (IL-1β and IL-18), and this phenomenon is mediated through the methylation of the apoptosis-associated protein caspase gene (ASC) and the downstream NLRP3 inflammasome^[66]^. Regular exercise has been shown to reduce inflammation, with evidence from epidemiological studies and randomized control trials demonstrating inverse associations between physical activity and markers of low-grade systemic inflammation^[67]^. Exercise activates molecular signals that may circumvent defects in insulin signaling in the skeletal muscle and increases the mitochondria of skeletal muscle, which is associated with an improvement of the insulin sensitivity in the skeletal muscle, thus improving the age-related effects of T2D^[68]^. Additionally, exercise halts telomere shortening, mitochondrial dysfunction, cellular senescence, and the production of SASP factors^[67]^.

## CONCLUSION

The number of older people continues to increase steadily. Consequently, there is an urgent need for therapies that can combat the effects of aging on the cardiovascular system. New insights into the mechanism of aging-related cellular dysfunction have paved the way for new therapies with an evolving body of basic research evidence. Ongoing and future translational studies are expected to demonstrate clinical evidence for the efficacy of new therapeutic strategies that mitigate age-related cardiac dysfunction. In addition to new pharmacological strategies, lifestyle modifications and exercise have been shown to modulate the cellular effects of aging and reduce the magnitude of inflammaging and associated cardiovascular disease. Current evidence suggests that age-related cardiovascular effects could become a treatable condition in the future.

## Data Availability

Not applicable.
